# Traditional Chinese medicine Danggui Shaoyao San for the treatment of Alzheimer's disease: A Protocol for Systematic Review

**DOI:** 10.1097/MD.0000000000019669

**Published:** 2020-04-10

**Authors:** Yu You, Xinglong Liu, Yanyan You, Dan Liu, Chunjiang Zhang, Yunhui Chen, Tiane Zhang

**Affiliations:** a Chengdu University of Traditional Chinese Medicine, No. 1166 Liutai Dadao Avenue, Wen Jiang District; b West China Hospital, Sichuan University, Wu Hou District, Chengdu, Sichuan, China.

**Keywords:** Alzheimer's disease, Danggui Shaoyao San, meta-analysis, protocol, randomized controlled trials, systematic review

## Abstract

**Background:**

Alzheimer's disease (AD) is the most common cause of dementia. Traditional Chinese formula Danggui Shaoyao San (DSS) has been considered a potential therapeutic approach for AD. However, no systemic review regarding its efficacy and safety has been conducted. Herein, we propose a *protocol* for the study that aims to evaluate the efficacy and safety of DSS in patients with AD.

**Methods:**

Sixteen electronic databases including PubMed, EMBASE, Cochrane database, Web of science, Chinese National Knowledge Infrastructure, VIP, Wanfang database, China Biomedical Literature Database, Chinese Clinical Trial Registry System, Koreanstudies Information Service System, Oriental Medicine Advanced Searching Integrated System, Research Information Sharing Service, DBpia, Korean Traditional Knowledge Portal, Japanese CiNii databases and J-STAGE databases will be searched from the inception up to February 29, 2020. Randomized controlled trials (RCTs) that meet the pre-specified eligibility criteria will be included. RevMan software (V.5.3.5) will be used to perform data synthesis following data extraction and publication risk assessment. Subgroup and sensitivity analysis will be performed according to the condition of included RCTs. The primary outcomes include changes in the Mini-Mental State Examination (MMSE), Alzheimer's Disease Assessment Scale-cognitive subscale (ADAS-Cog), and Activities of Daily Living scale (ADL). Additional outcomes are clinical effective rate and adverse event rate. The Grading of Recommendations Assessment, Development and Evaluation system will be used to assess the strength of the evidence.

**Results:**

This study will provide a well-reported and high-quality synthesis of RCTs on the efficacy and safety of DSS for the treatment of AD.

**Conclusion:**

This systematic review protocol will be helpful for providing evidence of whether DSS is an effective and safe therapeutic approach for patients with AD.

**Ethics and dissemination:**

Ethical approval is not necessary as this protocol is only for systematic review and does not involve privacy data or conduct an animal experiment. This protocol will be disseminated by a peer-review journal or conference presentation.

**Systematic review registration:**

PROSPERO CRD42020150450.

## 1 Introduction

Alzheimer's disease (AD) is the most common cause of dementia worldwide and may afflict about 13.8 million people age 65 and older by 2050.^[1]^ Its multifactorial pathogenesis remains elusive and may ascribe to genetic mutation, Aβ deposition, aberrant aggregation of Tau protein, intermediate neurons and network abnormalities, synaptic damage, mitochondrial dysfunction, endoplasmic reticulum stress function, cell autophagy, neuroinflammation and enteric dysbacteriosis.^2^,^3^,^4^,^5^,^6^,^7^ Albeit with pains-taking decades of drug research and development, currently prescribed medications yield slight and temporary improvement and disease-modifying treatment for AD is still unavailable.^[1]^

Danggui Shaoyao San (DSS), also known as Toki-shakuyaku-san (TJ-23) in Japan and Dangguijakyak-san (DJS) in South Korea, is a classic traditional Chinese medicine (TCM) formula. It was first recorded in the *Synopsis of Golden Chamber* written by Zhongjing Zhang (150–219 AD) and originally designed for halting gynecological problems. Its therapeutic effect on AD was first reported by Japan researchers in the 1980s and considerable attentions have been drawn to it as a potential therapeutic agent for AD since then.^[8]^ Data mining in the medical records revealed DSS has become one of most frequently used formulas for patient with AD in China.^[9]^ The efficacy of DSS for AD seems optimistic and it has been claimed as ‘New Hope for AD.^[8]^

However, from the perspective of evidence-based medicine, the hierarchy of evidence is core and systematic review of high-quality randomized controlled trials (RCTs) can provide clear evidence regarding the benefits of certain healthcare intervention.^[10]^ To the best of our knowledge, no systematic review on the RCTs of DSS for AD has been reported. Herein, a critical appraisal of the available evidence is warranted, and we would propose a protocol for a systematic review to evaluate the evidence of DSS's efficacy and safety for treating patients with AD.

## 2 Methods

### 2.1 Study registration

This systematic review protocol has been registered on PROSPTERO (www.crd.york.ac.uk/prospero/) with number CRD42020150450. Ethical approval is unnecessary because this study only involves the data of previous studies.

### 2.2 Eligibility criteria

#### 2.2.1 Type of study

Only RCTs can be included. Observation studies, animal research, case report, review, and meta-analysis are excluded.

#### 2.2.2 Participants

Patients diagnosed with *AD* (using any recognized diagnostic criteria, such as Diagnostic and Statistical Manual of Mental Disorders (DSM-IV), or Recommendations from the National Institute on Aging-Alzheimer's Association work groups on diagnostic guidelines for AD (NIA-AA), or Chinese Guidelines for the Diagnosis and Treatment of Alzheimer's Disease or Other Dementia. There is no restriction on the age, gender, nationality and nation of the patient and the duration and severity of the disease. Patient with

1) Lewy body, vascular dementia, frontotemporal, or any other forms of dementia; and
2) other disorders such as Parkinson disease, traumatic brain injury, stroke, and cancer that may impact cognitive function will be excluded.

#### 2.2.3 Interventions

AD patients treated with DSS with or without combining conventional WM treatments. No restriction regarding conventional WM regimen.

#### 2.2.4 Comparison

AD patients treated with the same conventional WM regimen as intervention group in the same original study. No restriction regarding conventional WM treatment regimen.

#### 2.2.5 Outcomes

The primary outcomes include changes in the Mini-Mental State Examination (MMSE), Alzheimer's Disease Assessment Scale-cognitive subscale (ADAS-Cog), and Activities of Daily Living (ADL) scale. Additional outcomes are clinical effective rate and adverse event rate.

#### 2.2.6 Language

No restriction.

### 2.3 Information source

We will search the following 16 databases from the inception to February 29, 2020 electronically, including PubMed, EMBASE, Cochrane Database, Web of Science, Chinese National Knowledge Infrastructure, VIP, Wanfang database, China Biomedical Literature Database, Chinese Clinical Trial Registry System, Koreanstudies Information Service System, Oriental Medicine Advanced Searching Integrated System, Research Information Sharing Service, DBpia, Korean Traditional Knowledge Portal, Japanese CiNii databases, and J-STAGE databases.

### 2.4 Search strategy

Two reviews will search the literature independently with cross-check. Any inconsistency will be solved by a third reviewer. Manual search will be performed if relevant literatures are found in the included studies. The electronic search will be conducted using a combination of following keywords

AD, Alzheimer, Alzheimer's disease, dementia, senile dementia, mild cognitive impairment, cognitive impairment, cognitive disorders, neurodegeneration, primary senile degenerative, cognitive senile degenerative, cognitive dysfunction, neurocognitive disorder, mild neurocognitive, cognitive decline, danggui shaoyao san, Toki-shakuyaku-san, danggui-jakyak-san, Dangguijakyak San, Tokishakuyakusan, Dangguijakyak-san, DSS, TJ-23, DJS, randomized controlled trial, controlled clinical trial, randomized, randomly, trails and RCT. Manual searches will also be conducted to identify the extra studies from the reference list. The search strategy for PubMed is presented in Table 1 and the strategy will be modified upon the requirement of other databases.

**Table 1:**
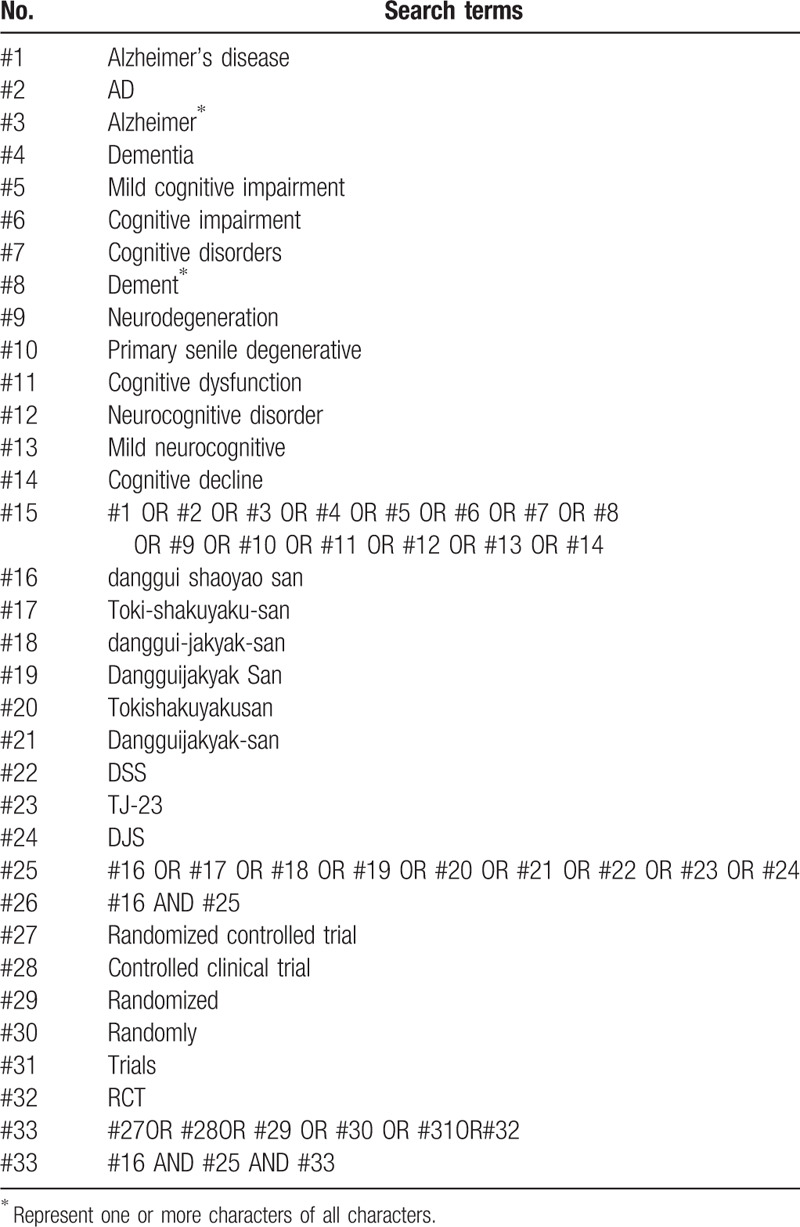
Search strategy for the PubMed.

### 2.5 Data collection and analysis

#### 2.5.1 Study selection

**Figure 1:**
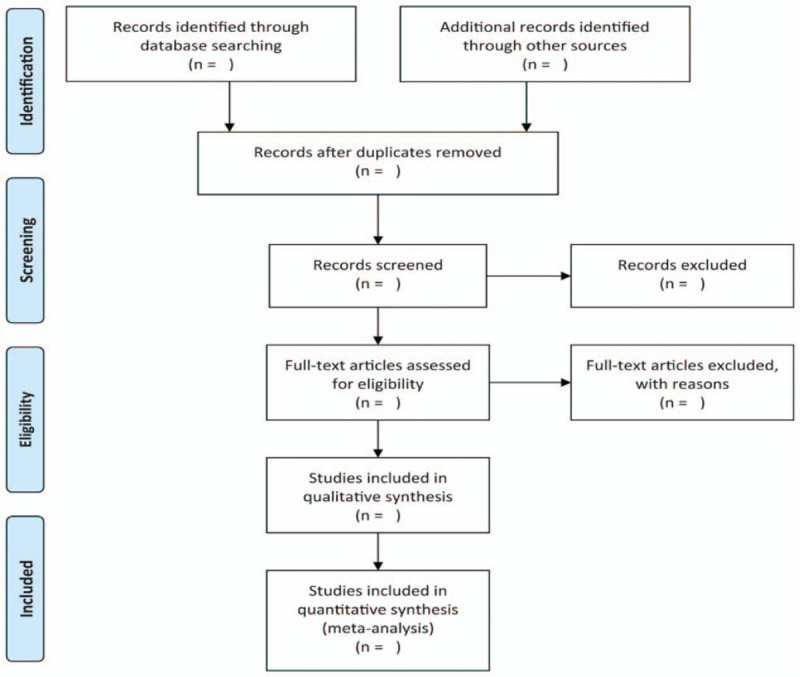
Flowchart of study selection.

Two reviewers will perform literature screening, study selection, and data extraction independently. The literature obtained will be imported into EndnoteX9 to screen the title and abstract, the duplications and studies failing to meet the pre-specified inclusion criteria will be excluded. After reading the full text of the remained literature and discussing within the group, the final included studies will be determined. The corresponding author of original RCT will be contacted when the full text is unavailable. Disagreements will be solved by consulting a third-party arbitrator or discussing within a group. The entire process of study selection is presented in the PRISMA flow diagram (Fig. 1).

#### 2.5.2 Data extraction and management

Two reviewers will independently extract data with a pre-specified standard form, including general information (title, serial number, first author, location of the RCTs, publication name, and published date), participants (age, gender, duration and severity of illness, and number), interventions of trial and control group (study design, sample size, details of randomization, blinding, allocation, intervention approach and duration), major outcomes, and minor outcomes. Inconsistency between two reviewers will be solved by a third reviewer.

#### 2.5.3 Risk of bias in included studies

Two reviews will assess the risk of publication bias for every included RCT with the Cochrane Risk of Bias Tool independently in terms of seven items, including random sequence generation, allocation concealment, blinding of participants and researchers, incomplete outcome data, selective reporting bias, and other bias. Each item will be graded as high, unclear, or low risk of bias. Inconsistency will be solved by consultation with a third reviewer.

#### 2.5.4 Measurement of treatment effect

Two reviewers will analyze the data independently using RevMan 5.3. Risk ratio (RR) with 95% confidence interval (CI) will be adopted for the dichotomous data, whereas the mean difference or standardized mean difference with 95% CI will be utilized for the continuous data.

#### 2.5.5 Management of missing data

If required data is unclear, missing or difficult to be obtained reliably, the corresponding author of the original RCT will be contacted by E-mail or telephone. If data is still unattainable, the study concerned will be excluded from the data analysis.

#### 2.5.6 Assessment of reporting biases

A funnel plot will be performed to assess any publication bias when more than 10 RCTs are included. In additional, Egger regression and Begg correlation test will also be performed to identify the funnel plot asymmetry.

#### 2.5.7 Assessment of heterogeneity

The Cochrane *I* ^2^ and Q tests will be applied to evaluate the heterogeneity with the cut-off value of *I* ^2^ = 50. If *I* ^2^ > 50% and/ or *Q* test <0.10, the heterogeneity will be deemed significant.

#### 2.5.8 Data synthesis

In line with the Cochrane guideline, a fixed-effect model will be utilized to pool and analyze the outcome data if *I* ^2^ < 50, and a random-effect model will be employed if *I* ^2^ ≥ 50. Subgroup analysis or meta-regression will be performed to assess the potential sources and present reasonable explanations for the heterogeneity.

#### 2.5.9 Sensitivity analysis

Sensitivity analysis will be applied to evaluate the stability of the pooled results of included RCTs according to the methodological quality, sample size and missing data.

#### 2.5.10 Grading the quality of evidence

The Grading of Recommendations Assessment, Development and Evaluation (GRADE) guidelines will be utilized to grade the quality of evidence as very low, low, moderate, or high.

## 3 Discussion

AD is a deleterious neurodegenerative disorder that characterized with progressive cognitive decline, functional impairment, neuropsychiatric signs and behavioral changes. Its complex multifactorial pathologies hinder the development of medical breakthroughs and none approaches are available to prevent and ameliorate AD. It has laid burgeoning obstacles on socioeconomic costs. DSS is a traditional Chinese medical formula and composed of *Angelica sinensis* (*Oliv*.) Diels (Umbelliferae), *Paeonia lactiflora* Pall. (Paeoniaceae), *Ligusticum chuanxiong* Hort. (Umbelliferae), *Poria cocos* (Schw.) Wolf (Polyporaceae), *Atractylodes macrocephala* Koidz. (Compositae) and *Alisma orientalis* (Sam.) Juzep. (Alismataceae) in a ration of 3:16:8:4:4:8.^[11]^ A growing body of studies have shown that DSS can alleviate cognitive dysfunction of AD patients, indicating that it may be a useful therapeutic agent for AD. This formula has presented anti-inflammatory and antioxidant activities, attenuated aberrant accumulation of Aβ and hyperphosphorylated Tau protein, reduced cell apoptosis in the hippocampus, mediated central monoamine neurotransmitters, ameliorated dysfunctional central cholinergic and dopaminergic nervous systems, and modulated the expression of estradiol.^12^,^13^,^14^,^15^,^16^

However, currently no systematic review and meta-analysis have been conducted regarding the efficacy and safety of DSS for the treatment of patient with AD. In this systematic review, all potential RCTs regarding the DSS for the treatment of AD will be fully considered and synthesized without language or publication restrictions. The findings of this study may yield helpful evidence for the clinicians and investigators concerned in decision-making process about the efficacy and safety of DSS for patients with AD.

## Author contributions

**Conceptualization:** Yunhui Chen, Yu You, Xinglong Liu, Tiane Zhang.

**Data curation:** Yunhui Chen, Yu You, Xinglong Liu.

**Formal analysis:** Yunhui Chen, Yanyan You, Dan Liu.

**Funding acquisition:** Yunhui Chen.

**Investigation:** Yu You, Yanyan You, Yunhui Chen, Xinglong Liu.

**Methodology:** Yunhui Chen, Yanyan You, Xinglong Liu.

**Project administration:** Yunhui Chen, Tiane Zhang.

**Supervision:** Tiane Zhang.

**Validation:** Chunjiang Zhang, Tiane Zhang.

**Writing – original draft:** Yunhui Chen, Yu You, Xinglong Liu, Tiane Zhang.

**Writing – review & editing:** Yunhui Chen, Yu You, Xinglong Liu, Tiane Zhang.

## References

[1] Alzheimer's Association. 2019 Alzheimer's disease facts and figures. Alzheimer's Dement 2019;15:321–87. 10.1016/j.jalz.2011.02.004 21414557

[2] Michael A Sugarman Michael L Neuropsychiatric symptoms and the diagnostic stability of mild cognitive impairment. J Alzheimers Dis 2018;62:1841–55. 29614641 10.3233/JAD-170527 PMC6548196

[3] Caroline VC Christine VB Kristel S The genetic landscape of Alzheimer disease: clinical implications and perspectives. Genet Med 2016;18:421–30. 26312828 10.1038/gim.2015.117 PMC4857183

[4] Rajmohan R Reddy PH Amyloid beta and phosphorylated tau accumulations cause abnormalities at synapses of Alzheimer's disease Neurons. J Alzheimers Dis 2017;57:975–99. 27567878 10.3233/JAD-160612 PMC5793225

[5] Anju S Ritushree K Luciano S Oxidative stress: a key modulator in neurodegenerative diseases. Molecules 2019;24:1583. 10.3390/molecules24081583 PMC6514564 31013638

[6] Inelia M Leonardo GM Cristóbal CT Neuroinflammation in the pathogenesis of Alzheimer's disease. A rational framework for the search of novel therapeutic approaches. Front Cell Neurosci 2014;8:112. 24795567 10.3389/fncel.2014.00112 PMC4001039

[7] Liu RQ Zhang TE Wang YQ Intestinal flora and Alzheimer's disease: research progress. Chin J Microecol 2019;31:112–5.

[8] Fu X Wang Q Wang Z Danggui-Shaoyao-San: new hope for Alzheimer's disease. Aging Dis 2015;7:502–13. 27493835 10.14336/AD.2015.1220 PMC4963193

[9] Li Q Cui YW Zhang YL Clinical literature research on TCM treatment on Alzheimer's disease. Liaoning J Trad Chin Med 2012;7:1207–10.

[10] Higgins JPT Green S Chapter 1: Introduction. In: *Cochrane Handbook for Systematic Reviews of Interventions* Version 5.1.0 [updated March 2011]. The Cochrane Collaboration, 2011. Available from www.handbook.cochrane.org.

[11] Zhang ZJ Chapter 20: Differentiation and Treatment of Disease during Pregnancy and Chapter 22 Differentiation and Treatment of Miscellaneous Gynecological Disease. In: *Synopsis of Golden Chamber*. Publishing House of Ancient Chinese Medical Books, 2019.

[12] Hao XY Luo S Cheng SY Effect of Danggui Shaoyaosan on Copper Ion-mediated Aβ Aggregation in AD Cell Model. Chin J Exp Trad Med Form 2019;25:45–51.

[13] Liu MY Huang DH Yan XF Effects of modified danggui shaoyao powder on expression of cerebral inflammatory Cytokines, CD45 and phosphorylated protein tau induced by amyloid beta 42 in rats. J Guang Univ Trad Chin Med 2013;30:357–62. 440-442.

[14] Zeng Y Xing ZZ Mei HF Effect of Danggui Shaoyao San and its active composition on learning and memory behaviors in cognitive impairment mice. J Guang Pharma Univ 2017;33:629–34.

[15] Zhong SZ Ma SP Hong ZY Anti-inflammation effect of Danggui Shaoyao San on Alzheimer's diseases. China J Chin Mater Med 2011;36:3155–60. 22375398

[16] Huang Y Hu ZY Yuan H Danggui-Shaoyao-San improves learning and memory in female SAMP8 via modulation of estradiol. Evid Based Complement Alternat Med 2014;2014:327294. 24757492 10.1155/2014/327294 PMC3976789

